# Effects of by-product feed-based silage on feeding, rumination, and excretion in growing Hanwoo heifers

**DOI:** 10.1186/s40781-014-0037-x

**Published:** 2015-01-27

**Authors:** Young-Il Kim, Sang Moo Lee, Youn Hee Lee, Myeon Lee, Do Young Choi, Wan Sup Kwak

**Affiliations:** Division of Food Bioscience, College of Health and Medical Life Sciences, Konkuk University, Chung-Ju, Chung-Buk 380-701 Korea

**Keywords:** Spent mushroom substrate, By-product feed, Rumination, Behavior, Hanwoo heifer

## Abstract

This study investigated the effects of feeding by-product feed (BF)-based silage on the behavior of growing Hanwoo heifers. Twelve Hanwoo heifers (13.2 months-old, 315 kg body weight; four heifers per pen) were assigned to three diets: a rice straw (RS) diet (concentrate mix and free access to RS), a RS and BF-based silage (RSBFS) diet (concentrate mix and free access to RS and BF-based silage), and a BF-based silage (BFS) diet (concentrate mix and free access to BF-based silage). Behavior was recorded for 5 days using camcorders. Compared to the RS group, the BFS group showed 21.7% higher dry matter intake, shorter feeding, rumination, and chewing times, as well as longer resting time (p < 0.05). Although all groups exhibited similar drinking, urination, and defecation frequencies, the BFS group exhibited higher feeding rates, rumination efficiency, and chewing efficiency than the RS group (p < 0.05). Compared to the BFS group, the RSBFS group showed higher peNDF_8.0_ intake (15.2% vs. 25.0% dry matter intake), longer feeding and sitting times, lower defecation frequency (p < 0.05), and similar rumination efficiency. In conclusion, complete replacement of conventional RS with BF-based silage reduced rumination and chewing activity in growing Hanwoo heifers, and BF-based silage feeding with large-particle straw is an effective approach in improving heifer behavior.

## Background

Demand for increased development of cheap domestic by-product feed (BF)-based roughage has increased in proportion to higher imported roughage and hay prices. Feeding of growing beef cattle with high quality roughage is an effective method of well-marbled beef [1]. Specifically, beef steers fed high quality timothy hay [2,3], during the growing period show improved growth and meat quality, and replacement of low quality rice straw (RS) with proteinaceous timothy and alfalfa hay has been shown to increase body weight gain and produce well-marbled beef [4,5]. However, as a limitation, the price of timothy hay is twice that of RS.

In our previous study [6], cheap and high quality BF-based roughage was successfully manufactured by ensiling spent mushroom substrate (SMS), recycled poultry bedding (RPB), rice bran, minimal straw with added molasses, and highly cellulolytic microbes, which were isolated from the SMS [7]. The silage exhibited favorable ensiling characteristics as well as higher degradability of dry matter (DM) and crude protein (CP) compared to RS or ryegrass straw [6]. Substitution of conventional RS with BF-based silage, which has a physically effective neutral detergent fiber (peNDF)_1.18_ of approximately 35%, did not significantly affect the rumination behavior of Hanwoo steers [8]. However, no information is yet available on the effects of BF-based silage on Hanwoo heifers.

A proper dietary level of peNDF is required to maintain the ruminal pH above 6.2. There are two types of peNDF, peNDF_8.0_ and peNDF_1.18_, and little information is available on which peNDF is more reliable for predicting rumination behavior in growing beef cattle.

This study was attempted to determine the effects of BF-based silage on feeding, rumination, resting, and excretion in growing Hanwoo heifers as well as the reliability of dietary peNDF_8.0_ and peNDF_1.18_ for predicting rumination behavior in growing beef cattle.

## Methods

### Animals and treatments

All animal care protocols were approved by the Konkuk University Institutional Animal Care and Use Committee. Twelve Hanwoo heifers with a mean age of 13.2 months and a mean body weight (BW) of 315 ± 1 kg were allocated to three pens (four heifers per pen). The area of each pen was 48 m^2^ (4 m × 12 m). Animals were fed one of three diets: a RS diet (concentrate mix and free access to RS), a RS and BF-based silage (RSBFS) diet (concentrate mix and free access to RS and BF-based silage), and a BF-based (BFS) silage diet (concentrate mix and free access to BF-based silage). Feed was supplied twice a day (07:00 and 18:00). All animals were fed 4.7 kg/d concentrate mix (normal commercial formulated feed) on a restricted basis, 0.45 kg/d alfalfa hay, with free access to fresh water. All heifers were acclimatized to their diets and housing for over 1 month before the experiment.

### Manufacturing BF-based silage

Fresh SMS was collected from a local oyster mushroom (*Pleurotus ostreatus*) farm. The original mushroom substrate consisted of 70% sawdust, 15% rice bran, and 15% corncobs. BF-based silage was manufactured as described by [6]. SMS (50%) was mixed with RPB (21%), cut ryegrass straw (15%), rice bran (10.8%), molasses (2%), bentonite (0.6%), and a microbial additive (0.6%), followed by ensilation for over 2 weeks in two layers of polyvinyl bags placed in a 1-ton capacity plastic bag. The chemical compositions of feeds are presented in Table 1. BF-based silage contained more CP and ether extract (EE) but less NDF and acid detergent fiber (ADF) than RS.

**Table 1:** **Particle size distribution, physical effectiveness factors (pef), and physically effective fiber (peNDF) content of the feeds**

**Item**	**Rice straw**	**BF-based silage** ^**1)**^	**Alfalfa hay**	**Concentrate mix**
% dry matter (DM) retained on sieves				
Above 19.0 mm	97.8	10.3	45.8	0.8
8.0–19.0 mm	1.2	8.2	10.7	53.9
1.18–8.0 mm	0.9	54.6	26.2	45.1
Below 1.18 mm	0.1	26.9	17.3	0.2
Pef_8.0_	99.0	18.5	56.5	53.9
pef_1.18_	99.9	73.1	82.7	39.4
peNDF_8.0_, % of DM	71.2	9.4	27.7	17.6
peNDF_1.18_, % of DM	71.8	37.0	40.6	12.9

^1)^BF-based silage was a by-product feed-based silage, which was composed of 50% spent mushroom substrate, 21% recycled poultry bedding, 15% ryegrass straw, 10.8% rice bran, 2% molasses, 0.6% bentonite, and 0.6% microbial culture, and was ensiled for over 2 weeks.

### Particle size determination

Particle sizes of the experimental feeds were measured using a Penn State Particle Separator, according to Kononoff and Heinrichs [9]. The separator consisted of three sieves (1.18, 8, and 19 mm) that could separate feeds into four types by particle size. For the physical effectiveness factor (pef), the proportion of particles larger than 8 mm was considered to be pef_8.0_, according to Lammers et al. [10], and the proportion of particles larger than 1.18 mm was considered to be pef_1.18_, according to Kononoff and Heinrichs [9]. The peNDF_8.0_ and peNDF_1.18_ were calculated by multiplying the NDF percentage by pef_8.0_ and pef_1.18_, respectively. For calculation of the concentrate mix pef_1.18_, the ratio of the corn flake portion to the other portions was determined. The pef values for the corn flake portion and the other concentrate mix pellets were 0.8 and 0.3, respectively, according to Metens [11]. The particle sizes of RS, alfalfa hay, BF-based silage, and concentrate mix used in this experiment are presented in Table 1. Compared to RS, BF-based silage had much smaller particles and much lower pef and peNDF values.

### Behavioral observation methods and analysis

Prior to the experiment, the animals were adapted to night lighting for 10 days. Three camcorders (SVR-450, Samsung, Korea) were installed outside of the pen edge, and data were recorded for 120 h at each of the three pens. Feeding, rumination, and resting times, together with frequencies of defecation and urination, were measured at 1-min intervals and recorded on plotting paper.

Feed intake was calculated by measuring the difference between the supplied and remaining amounts of feed, and the remaining ort was retrieved and measured before the next morning’s feeding. Chewing time was calculated by summing feeding and rumination times, and feeding, rumination, and chewing efficiencies were calculated by dividing voluntary DM intake by the respective feeding, rumination, or chewing times.

### Chemical analysis

Representative samples of the test feeds were collected and stored at −20°C for later analysis. Immediately before the analysis, all samples were dried, ground, and passed through a 1-mm screen using a Sample mill (Cemotec, Tecator, Sweden). The DM was determined by drying the samples at 65°C for 48 h to constant weight. CP (N × 6.25) and EE contents were determined using AOAC [12] methods. Crude ash was determined by heating samples at 600°C for 3 h. Ash-free NDF and ADF were determined according to Van Soest et al. [13]. Non-fibrous carbohydrate content was calculated as 100 - (NDF % + CP % + EE % + crude ash %). As shown in Table 2, CP content of BF-based silage was 12.0%, which was 3.3-fold higher than that of RS, and the NDF of BF-based silage was 21.3% lower than that of RS.

**Table 2:** **Chemical compositions of the feeds fed to growing Hanwoo heifers**

**Item**	**Rice straw**	**BF-based silage** ^**1)**^	**Alfalfa hay**	**Concentrate mix**
	%, DM basis			
Dry matter	85.0	58.3	83.3	87.9
Crude protein	3.6	12.0	14.7	16.4
Ether extract	1.1	3.9	1.2	2.5
Crude ash	8.3	11.5	9.9	8.3
Neutral detergent fiber	71.9	50.6	49.1	32.7
Acid detergent fiber	42.5	38.9	36.9	19.5
Non-fibrous carbohydrate	15.1	17.1	25.1	40.1

^1)^BF-based silage was a by-product feed-based silage, which was composed of 50% spent mushroom substrate, 21% recycled poultry bedding, 15% ryegrass straw, 10.8% rice bran, 2% molasses, 0.6% bentonite, and 0.6% microbial culture, and was ensiled for over 2 weeks.

### Statistical analysis

Data were subjected to one-way analysis of variance (ANOVA) using the general linear model (GLM) procedure [14]. Comparisons of means between RS, RSBFS, and BFS treatments were made using Tukey’s multiple range test [14], and significance was set at p < 0.05.

## Results and discussion

### Daily feed intake

Daily feed intake of DM, NDF, and peNDF is presented in Table 3. Upon feeding with concentrate mix and alfalfa hay in equal amounts, daily roughage intake per head was 0.15 kg higher in the RSBFS group and 1.42 kg higher in the BFS group compared to the RS group. This increased feed intake is partly attributed to the fact that BF-based silage particles were smaller than those of RS, and it is known that small particle size can increase feed intake [15,16]. Martz and Belyea [17] reported that small particle size stimulates rapid ruminal passage of digesta and increases feed DM intake. Kim et al. [18] also reported that RS with a small particle size increases feed intake, and Kononoff and Heinrichs [9] reported that feed DM intake increases linearly as feed particle size decreases. Other reasons for increased feed intake may be favorable palatability and/or high digestibility of BF-based silage [6].

**Table 3:** **Effects of feeding by-product feed-based silage on feed dry matter (DM) and neutral detergent fiber (NDF) intakes in growing Hanwoo heifers**

**Item**	**Diet** ^**1)**^ **with**		
	**RS**	**RSBFS**	**BFS** ^**2)**^
Feed DM intake, kg/d	6.53	6.68	7.95
Concentrate mix	4.70	4.70	4.70
Alfalfa hay	0.45	0.45	0.45
Rice straw	1.38	0.93	-
BF-based silage	-	0.60	2.80
NDF intake, kg/d	2.75	2.73	3.17
Concentrate mix	1.54	1.54	1.54
Roughage	1.21	1.19	1.64
% of DMI	42.1	40.9	39.9
peNDF_8.0_ intake, kg/d	1.93	1.67	1.22
% of DMI	29.6	25.0	15.3
peNDF_1.18_ intake, kg/d	1.78	1.68	1.82
% of DMI	27.3	25.1	22.9

^1)^The three diets were as follows: RS (free access to rice straw); RSBFS (free access to rice straw and BF-based silage); and BFS (free access to BF-based silage).

^2)^BF-based silage (BFS) was a by-product feed-based silage, which was composed of 50% spent mushroom substrate, 21% recycled poultry bedding, 15% ryegrass straw, 10.8% rice bran, 2% molasses, 0.6% bentonite, and 0.6% microbial culture, and was ensiled for over 2 weeks.

NDF intake by the BFS group was 15% higher than that by the RS group. Feeding of the BFS group with BF-based silage resulted in increased feed NDF intake due to higher feed DM intake, although the NDF content of BF-based silage was 21.3% lower than that of RS. The proportion of NDF intake per DM intake was similar (40–42%) between treatments. These levels fully satisfied the minimum criterion of 25% dietary NDF, which is recommended for maintenance of normal ruminal pH [19]. In general, rumination time increases as NDF content increases, which helps to maintain a normal ruminal pH [20,21].

Intake of peNDF_8.0_ decreased by 13.9% in the RSBFS group and by 37.6% in the BFS group. The reduced peNDF_8.0_ intake per DM intake in the BFS group was due to the large difference in pef_8.0_ between RS and BF-based silage (99.0% vs. 18.5%, respectively). The 15.3% peNDF_8.0_ intake per DM intake was just above the minimal 15% level for maintenance of ruminal pH above 6.0, as suggested by Beauchemin et al. [22] and Zebeli et al. [23]. In the RSBFS group, peNDF_8.0_ intake per DM intake was higher than that of the BFS group.

The peNDF_1.18_ intake per DM intake (23–25%) decreased by 2–4% when hiefers were fed BF-based silage. It has been reported that peNDF_1.18_ intake per DM intake should be 30% [11] or 30–32% [24] for maintenance of ruminal pH above 6.2, whereas it should be at least 22.3% to maintain ruminal pH above 6.0 [11]. In the present study, 23–25% peNDF_1.18_ intake per DM intake in the BF-based silage-fed groups was sufficient to maintain ruminal pH above 6.0 but insufficient to maintain ruminal pH above 6.2. Consequently, for prediction of rumination behavior, dietary peNDF_8.0_ intake seemed to be a more reliable index than dietary peNDF_1.18_ intake during the rumen-developing period of growing cattle, as also reported by Zebeli et al. [25].

### Feeding, rumination, and resting

The effects of BF-based silage on feeding, rumination, chewing, and resting are presented in Table 4. The feeding times for dietary DM and NDF were longest in the RS group and shortest in the BFS group (p < 0.05). This phenomenon can be attributed to the smaller particle size of BF-based silage. Other studies also found that small particle size reduces time taken to eat [20,9]. BF-based silage feeding did not affect feeding time of dietary peNDF_8.0_, although it decreased (p < 0.05) feeding time of dietary peNDF_1.18_ in the BFS group.

**Table 4:** **Effects of by-product feed-based silage on feeding, rumination, chewing, and resting in growing Hanwoo heifers**

**Item**	**Diet** ^**1)**^ **with**			**SE**	**p-value**
	**RS**	**RSBFS**	**BFS** ^**2)**^		
	min/d				
Eating time	289.0^a^	230.8^b^	159.8^c^	17.6	0.0002
Per kg of DM	44.3^a^	34.6^b^	20.1^c^	2.6	0.0001
Per kg of NDF	105.2^a^	84.6^b^	50.3^c^	6.3	0.0001
Per kg of peNDF_8.0_	149.2	138.3	131.4	10.9	0.2978
Per kg of peNDF_1.18_	162.6^a^	137.7 ^a^	87.6 ^b^	10.1	0.0001
Ruminating time	458.3^a^	282.0^b^	317.5^b^	26.0	0.0002
Per kg of DM	70.2^a^	42.2^b^	39.9^b^	3.7	0.0001
Per kg of NDF	166.8^a^	103.4^b^	100.0^b^	9.1	0.0001
Per kg of peNDF_8.0_	237.0^a^	169.0^b^	261.2^a^	17.1	0.0012
Per kg of peNDF_1.18_	257.9^a^	168.3^b^	174.1^b^	14.9	0.0003
Chewing time^3)^	747.3^a^	512.8^b^	477.3^b^	31.7	0.0001
Per kg of DM	114.5^a^	76.8^b^	60.0^c^	4.6	0.0001
Per kg of NDF	271.9^a^	188.0^b^	175.0^b^	11.5	0.0001
Per kg of peNDF_8.0_	386.5^a^	307.2^b^	392.7^a^	19.8	0.0033
Per kg of peNDF_1.18_	420.5^a^	306.1^b^	261.7^b^	18.1	0.0001
Resting time	692.8^b^	927.3^a^	962.8^a^	31.3	0.0001
Standing time	791.8^a^	504.8^b^	668.0^a^	44.5	0.0004
Sitting time	648.2^b^	935.2^a^	772.0^b^	44.5	0.0004

^1)^The three diets were as follows: RS (free access to rice straw); RSBFS (free access to rice straw and BF-based silage); and BFS (free access to BF-based silage).

^2)^BF-based silage (BFS) was a by-product feed-based silage, which was composed of 50% spent mushroom substrate, 21% recycled poultry bedding, 15% ryegrass straw, 10.8% rice bran, 2% molasses, 0.6% bentonite, and 0.6% microbial culture, and was ensiled for over 2 weeks.

^3)^Chewing time = eating time + rumination time.

^a,b,c^Means with different superscripts within the same row are significantly different (p < 0.05).

Rumination time per dietary intake of DM, NDF, or peNDF_1.18_ was shorter in the BF-based silage groups than in the RS group (p < 0.05), despite higher dietary DM and NDF intakes. Okine and Mathison [26] reported that dietary NDF intake increases with rumination time. However, in the present study, BF-based silage feeding increased dietary NDF intake while reducing rumination time by 38–39% mainly due to decreased peNDF_8.0_ intake (up to 48%), which otherwise stimulates rumination activity. In the present study, the rumination time of 317.5 min/d in the BFS group is similar to the 322.5 min/d rumination time previously reported in Hanwoo steers (mean BW of 500 kg) fed a 40:60 ratio of RS and concentrate mix [27]. This level is also higher than the 245.8 min/d rumination time of early fattening Hanwoo steers (491 kg BW) fed 1.1 kg of RS (12%) and 7.6 kg of concentrate mix (88%) [28], whereas it is lower than the 451 min/d rumination time of growing Hanwoo steers (357 kg BW) fed 2.7 kg of RS (33%) and 5.3 kg of concentrate mix (67%) [29].

The values for chewing time (feeding time plus rumination time) were highest in the RS group and lowest in the BFS group (p < 0.05), and chewing time per dietary DM or NDF intake was also lowest in the BFS group (p < 0.05). Similarly, Mertens [11] reported that as feed intake increases, chewing time per DM intake decreases since the amount of time that animals are able to chew per day is limited. In addition, Beauchemin et al. [22] reported that as peNDF_1.18_ intake increases, chewing time per kg of peNDF_1.18_ decreases, as partially confirmed in the present study. We found that 15.3% dietary intake of peNDF_8.0_ in the BFS group suppressed rumination and chewing activity.

BF-based silage feeding resulted in a longer resting time compared to RS (p < 0.05). Standing and sitting times were similar between the RS and BFS groups, whereas the RSBFS group spent more time sitting compared to the BFS group (p < 0.05).

### Drinking and excretion

The effects of BF-based silage feeding on drinking and excretion are shown in Table 5. Complete replacement of RS with BF-based silage did not affect drinking, urination, or defecation frequency. The increased feed intake by heifers fed BF-based silage did not result in significant elevation of excretion frequency. Ryu et al. [30] reported that urination frequency varies with feed type and cattle age. In our study, the RSBFS group showed a significantly lower defecation frequency (p < 0.05) than the BFS group.

**Table 5:** **Effects of feeding by-product feed-based silage on drinking, urination, and defecation in growing Hanwoo heifers**

**Item**	**Diet** ^**1)**^ **with**			**SE**	**p-value**
	**RS**	**RSBFS**	**BFS** ^**2)**^		
Drinking frequency/d	10.3	7.0	6.8	1.7	0.1153
Urinating frequency/d	4.5	4.3	5.3	1.2	0.6829
Defecating frequency/d	5.5^ab^	3.5^b^	6.8^a^	1.1	0.0309

^1)^The three diets were as follows: RS (free access to rice straw); RSBFS (free access to rice straw and BF-based silage); and BFS (free access to BF-based silage).

^2)^BF-based silage (BFS) was a by-product feed-based silage, which was composed of 50% spent mushroom substrate, 21% recycled poultry bedding, 15% ryegrass straw, 10.8% rice bran, 2% molasses, 0.6% bentonite, and 0.6% microbial culture, and was ensiled for over 2 weeks.

^a,b,c^Means with different superscripts within the same row are significantly different (p < 0.05).

### Feeding rate, rumination efficiency, and chewing efficiency

The effects of BF-based silage on feeding rate, rumination efficiency, and chewing efficiency are presented in Table 6. The feeding rate was highest in the BFS group and lowest in the RS group (p < 0.05). Kim et al. [18] stated that feeding rate increases when the length of feeding time is shortened, and Jeon et al. [31] found that the feeding rate is high when using roughage with a small particle size. In the present study, rumination efficiency was high in the BF-based silage groups (p < 0.05). Lee et al. [29] reported that rumination efficiency varies with feed characteristics, animal age, and health status, and Balch [32] stated that rumination efficiency is a key factor in determining the physical characteristics of a feed.

**Table 6:** **Effects of feeding by-product feed-based silage on feeding rate, rumination efficiency, and chewing efficiency in growing Hanwoo heifers**

**Item**	**Diet** ^**1)**^ **with**			**SE**	**p-value**
	**RS**	**RSBFS**	**BFS** ^**2)**^		
Eating rate^3)^	1,365.8^b^	1,751.6^b^	3,016.6^a^	167.1	0.0001
Ruminating efficiency^4)^	857.6^b^	1,446.6^a^	1,516.7^a^	119.5	0.0007
Chewing efficiency^5)^	525.2^c^	788.4^b^	1,003.9^a^	50.2	0.0001

^1)^The three diets were as follows: RS (free access to rice straw); RSBFS (free access to rice straw and BF-based silage); and BFS (free access to BF-based silage).

^2)^BF-based silage (BFS) was a by-product feed-based silage, which was composed of 50% spent mushroom substrate, 21% recycled poultry bedding, 15% ryegrass straw, 10.8% rice bran, 2% molasses, 0.6% bentonite, and 0.6% microbial culture, and was ensiled for over 2 weeks.

^3)^Voluntary intake (DM g/d)/feeding time (h/d).

^4)^Voluntary intake (DM g/d)/rumination time (h/d).

^5)^Voluntary intake (DM g/d)/chewing time (h/d).

^a,b,c^Means with different superscripts within the same row are significantly different (p < 0.05).

Chewing efficiency was highest in the BFS group and lowest in the RS group (p < 0.05). Woodford et al. [20] and Ryu et al. [29] found that crushed and chopped feed with a small particle size reduces feeding time compared with feeds in their original form. Considering this, the increased chewing efficiency in the BF-based silage groups can be attributed to increased feeding rate and decreased feeding time. Therefore, the increase in feed DM intake by BF-based silage feeding can also be explained by improved feeding and chewing efficiencies. In a study by Lee et al. [33], wet total mixed ration feeding in a restricted or *ad libitum* manner resulted in elevated feed intake, followed by increases in feeding, rumination, and chewing efficiencies. These phenomena can improve the ruminal environment for enhanced animal productivity.

In conclusion, compared to RS, *ad libitum* BF-based silage feeding resulted in higher feed DM and NDF intake, shorter feeding, rumination, and chewing times, longer resting times (p < 0.05), as well as higher feeding, rumination, and chewing efficiencies (p < 0.05). Both groups exhibited similar frequencies of defecation, urination, and drinking. Compared to only *ad libitum* BF-based silage feeding, RS and BF-based silage together resulted in higher peNDF_8.0_ intake (15.2 vs. 25.0% of DMI, respectively), longer feeding and sitting times, lower defecation frequency (p < 0.05), and a similar rumination efficiency.

## Conclusions

Dietary peNDF_8.0_ intake level was a better predictor of rumination behavior in growing Hanwoo heifers than dietary peNDF_1.18_ intake, and the complete replacement of conventional RS with BF-based silage depressed rumination activity. BF-based silage feeding with large-sized particle straw is an effective way of improving the behavior pattern of growing Hanwoo heifers.
